# Identification of let-7a-2-3p or/and miR-188-5p as Prognostic Biomarkers in Cytogenetically Normal Acute Myeloid Leukemia

**DOI:** 10.1371/journal.pone.0118099

**Published:** 2015-02-03

**Authors:** Shi Jinlong, Fu Lin, Li Yonghui, Yu Li, Wang Weidong

**Affiliations:** 1 Medical Engineering Division, Hainan Branch, Chinese PLA General Hospital, Sanya, Hainan, China; 2 Medical Engineering Support Center, Chinese PLA General Hospital, Beijing, China; 3 Department of Hematology and Lymphoma Research Center, Peking University, Third Hospital, Beijing, China; 4 Department of Hematology, Chinese PLA General Hospital, Beijing, China; Queen’s University Belfast, UNITED KINGDOM

## Abstract

Cytogenetically normal acute myeloid leukemia (CN-AML) is the largest and most heterogeneous AML subgroup. It lacks sensitive and specific biomarkers. Emerging evidences have suggested that microRNAs are involved in the pathogenesis of various leukemias. This paper evaluated the association between microRNA expression and prognostic outcome for CN-AML, based on the RNA/microRNA sequencing data of CN-AML patients. High let-7a-2-3p expression and low miR-188-5p expression were identified to be significantly associated with longer overall survival (OS) and event free survival (EFS) for CN-AML, independently or in a combined way. Their prognostic values were further confirmed in European Leukemia Net (ELN) genetic categories. Also, in multivariable analysis with other known risk factors, high let-7a-2-3p and low miR-188-5p expression remained to be associated with longer OS and EFS. In addition, the prognostic value of these two microRNAs was confirmed in patients who received hematopoietic stem cell transplantation (HSCT). To gain more biological insights of the underlying mechanisms, we derived the genome-wide differential gene/microRNA signatures associated with the expression of let-7a-2-3p and miR-188-5p. Several common microRNA signatures indicating favorable outcome in previous studies were up-regulated in both high let-7a-2-3p expressers and low miR-188-5p expressers, including miR-135a, miR-335 and miR-125b and all members of miR-181 family. Additionally, common up-regulated genes included FOSB, IGJ, SNORD50A and ZNF502, and FOSB was a known favorable signature in AML. These common signatures further confirmed the underlying common mechanisms for these two microRNAs value as favorable prognostic biomarkers. We concluded that high let-7a-2-3p and low miR-188-5p expression could be potentially used as favorably prognostic biomarkers independently or in a combined way in CN-AML patients, whether they received HSCT or not.

## Introduction

Cytogenetically-normal acute myeloid leukemia (CN-AML) accounts for almost half of patients with AML[1]. Such patients usually fall into intermediate-risk cytogenetics, but show obviously heterogeneous prognosis clinically. Mutations and gene expression signatures have been used to distinguish and identify different prognostic subgroups[2,3]. Mutations of NPM1[4] and CEBPA[5] are associated with favorable outcome, whereas mutations of FLT3-ITD[6] and WT1[7] supply indication for adverse prognosis. Also, low expression of BAALC and ERG[8], high expression of LEF1[9] have shown to be favorable prognostic factors. Because of the sizeable proportion of patients that failed to existing therapies and high relapse rate, identification of new diagnostic and prognostic biomarkers is valuable for finding novel targets and developing novel therapies.

MicroRNA is a large family of highly conserved noncoding genes with a short single strand of 19 to 25 nucleotides in length, which are directly involved in many critical processes, including cell differentiation, apoptosis, proliferations and hematopoiesis [10–12]. Emerging data shows that many types of solid tumors and leukemias are accompanied with the dysregulation of microRNAs [13,14]. For their direct and indirect involvement in hematopoiesis, recently, more and more function studies and prognostic analysis were performed on various leukemias. A cluster of two miRNAs, miR-15a and miR-16–1, were firstly linked to be involved in chronic lymphocytic leukemia (CLL)[15]. The diagnostic and therapeutic impact about microRNAs was discussed in leukemias[16,17]. However, studies testing the prognostic impact of let-7a-2–3p and miR-188–5p expression have not been reported in CN-AML.

This paper tries to determine the prognostic impact of the expression of let-7a-2–3p and miR-188–5p in CN-AML patients. For the first time, it presented that high expression of let-7a-2–3p and low expression of miR-188 were significantly associated with better outcome, independently or in a combined way, for CN-AML patients whether they received hematopoietic stem cell transplantation (HSCT) or not. Their prognostic values were further confirmed in European Leukemia Net (ELN) genetic categories and multivariable analysis adjusting other known risk factors. To further explore the underlying biological mechanisms of why high let-7a-2–3p and low miR-188 expression were associated with improved outcome, genome-wide sequencing data of gene and microRNA was analyzed to reveal the aberrant changes of expression pattern. Differentially expressed genes/microRNAs were identified, according to the high and low expression of these two microRNAs. Interestingly, many genes/microRNAs were identified to be commonly up/down-regulated associated with high expression of let-7a-2–3p and low expression of miR-188–5p, including several known favorable prognostic biomarkers, which validated the potential value of these two microRNAs in outcome prediction. In conclusion, these findings may potentially be used in clinical application and inspire new strategies for diagnosis and therapy for CN-AML patients.

## Methods

### Patients and expression profiles of gene and microRNA

TCGA provided 200 clinically annotated adult de novo AML samples, and performed RNA sequencing for 179 samples and microRNA sequencing for 194 samples as previously reported[18]. In all these cases, sample numbers of CN-AML was 79 with microRNA sequencing and 74 with RNA sequencing, respectively. These sequencing data provided exact measure for expression levels. Also, detailed descriptions were provided for clinical and molecular characteristics. All these data can be publicly downloaded from the TCGA website, providing a consolidated support for statistical analysis. Written informed consent was obtained from all patients, which was approved by the human studies committee at Washington University[18].

### Statistical analysis

The main objective of the study was to evaluate the prognostic value of let-7a-2–3p and miR-188–5p expression in CN-AML. Firstly, median values of let-7a-2–3p and miR-188–5p expression were used to divide all patients into two groups, separately. Then, Kaplan-Meier method was used to estimate the association between microRNA expression levels and overall survival (OS) and event free survival (EFS), which were further validated with log-rank test. Definition of these clinical endpoints (OS, EFS) was described in [18].

To further investigate the association between microRNA expression levels and clinical, molecular features, the Fisher exact and Wilcoxon rank-sum tests were used to check the hypothesis testing for categorical and continuous variables, respectively. In addition, multivariable Cox proportional hazards models were used to study how these two microRNAs’ expression levels were associated with OS and EFS accompanying with other known risk factors. According to the two groups divided by microRNA expression levels, Student’s *t*-test was used to identify differentially expressed genes/microRNAs, the derived *p* values were corrected with multiple hypothesis of BH test (Benjamini& Hochberg). The statistic cutoffs were confirmed as absolute fold-change (FC) > 1.5 and the adjusted *P*-value < 0.05.

All analyses were performed on the platform of R 3.0.2 software package.

## Results

### Clinical and molecular characteristics associated with let-7a-2–3p and miR-188–5p expression

Most clinical and molecular features show no significant difference associated with different expression status of let-7a-2–3p and miR-188–5p. But it can be seen that high expression of let-7a-2–3p was associated with mutation of CEBPA (*P* = 0.03, Table 1), which can be seen as a biomarker for favorable prognosis in CN-AML [19]. This trend of association can also be seen in low miR-188–5p expressers (*P* = 0.057, Table 1).

**Table 1:** Patients’ characteristics according to let-7a-2–3p and miR-188–5p expression levels.

Characteristic	High let7a-2–3p, n = 39	Low let-7a-2–3p, n = 40	P	High miR-188, n = 39	Low miR-188, n = 40	P
Age, years			0.357			0.587
Median	57	58		62	56	
Range	21–88	25–83		23–88	21–76	
WBC			0.736			0.7
Median	18.7	40.45		134.2	26.3	
Range	0.6–297.4	1.4–298.4		1.4–298.4	0.6–297.4	
BM Blast %			0.687			0.548
Median	71	75		74	73.5	
Range	33–100	30–98		30–98	32–100	
PB Blast %			0.131			0.015
Median	53	36		32	55	
Range	0–98	0–88		0–90	0–98	
FLT3, no (%)			0.31			1
ITD-Mutated	10 (26)	13 (33)		12 (31)	11 (27)	
TKD-Mutated	2 (5)	7 (17)		4 (10)	5 (13)	
Wild Type	27 (69)	20 (50)		23 (59)	24 (60)	
NPM1, no (%)			0.367			0.821
Mutated	20 (51)	25 (63)		23 (59)	22 (55)	
Wild Type	19 (49)	15 (37)		16 (41)	18 (45)	
CEBPA, no (%)			0.03			0.057
Mutated	7 (18)	1 (2)		1 (3)	7 (17)	
Wild Type	32 (82)	39 (98)		38 (97)	33 (83)	
ELN genetic groups			0.5			0.65
Favorable	19	16		16	19	
Intermediate-I	20	24		23	21	
DNMT3A, no (%)			0.359			0.494
Mutated	13 (33)	18 (45)		17 (44)	14 (35)	
Wild Type	26 (67)	22 (55)		22 (56)	26 (65)	
IDH2, no (%)			0.737			0.193
Mutated	4 (11)	6 (15)		7 (18)	3 (8)	
Wild Type	35 (89)	34 (85)		32 (82)	37 (92)	

### Prognostic impact of let-7a-2–3p and miR-188–5p expression

**Fig 1:**
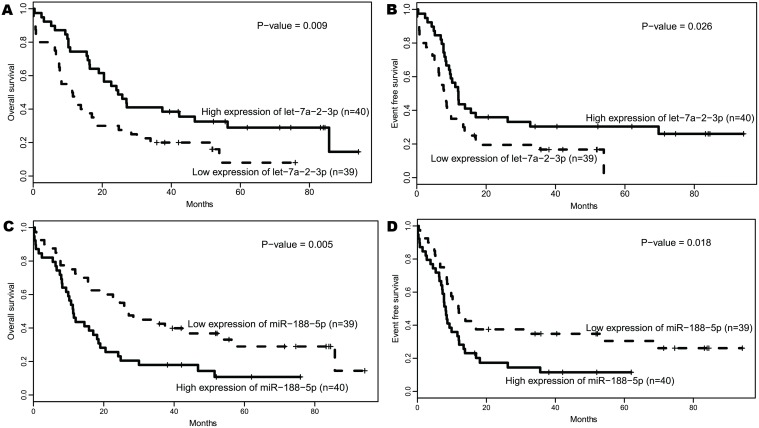
Survival of CN-AML patients according to the expression of let-7a-2–3p and miR-188–5p.

Using median expression as the cutoff, patients were divided into high and low expression groups. High let-7a-2–3p expressers had a longer OS (*P* = 0.009, Fig. 1A) and EFS (*P* = 0.026, Fig. 1B) than low ones, while low expression of miR-188–5p showed a significant association with longer OS (*P* = 0.005, Fig. 1C) and EFS (*P* = 0.018, Fig. 1D) than high expression. In addition, mean OS, EFS and relapsing time associated with high or low expression groups of let-7a-2–3p and miR-188–5p were compared separately, results showed there did exist significant difference between high and low expression of these two microRNAs, respectively. (For let-7a-2–3p, OS: *P* = 0.0045, EFS: *P* = 0.016, relapsing days: *P* = 0.029. For miR-188–5p, OS: *P* = 0.012, EFS: *P* = 0.027, relapsing days: *P* = 0.047, Table 2).

**Table 2:** Treatment response according to let-7a-2–3p and miR-188–5p expression.

	High let-7a, n = 39	Low let-7a, n = 40	p	High miR-188, n = 39	Low miR-188, n = 40	p	High let-7a and low miR-188, n = 27	High let-7a and/or low miR-188, n = 27	p
OS, months			0.0045			0.012			<0.001
Median	24.45	11.30		11.4	27		32.25	11.5	
Range	0.4–94.2	0.1–75.9		0.1–75.9	0.4–94.2		0.4–94.2	0.1–75.9	
EFS, months			0.016			0.027			<0.001
Median	11.95	8.20		8.25	11.9		14.75	8.3	
Range	0.4–94.2	0.1–53.9		0.1–71.4	0.4–94.2		0.4–94.2	0.1–62	
Relapse days			0.029			0.047			0.006
Rate, %	69	58	0.14	68	59	0.23	66.7	61.5	0.55
Median	338	248		252	310		364	254.5	
Range	130–2119	82–1082		82–1082	130–2119		130–2119	82–1082	

**Fig 2:**
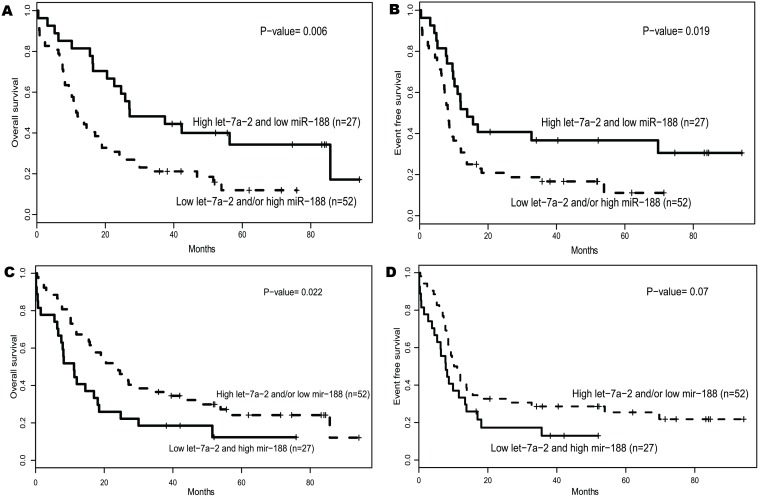
Survival of CN-AML patients according to the combination of let-7a-2–3p and miR-188–5p expression levels.

To further explore the prognostic impact of these two microRNAs, we investigated the combination of them. Firstly, patients with both higher expression of let-7a-2–3p and lower expression of miR-188–5p were compared with others to show the power of the combination of the 2 signatures for favorable prognosis. The combination of 2 expression signatures showed a significant longer OS (*P* = 0.006, Fig. 2A), EFS (*P* = 0.019, Fig. 2B) and relapsing days (*P* = 0.006, Table 2) than that of low let-7a-2–3p expressers and/or high miR-188–5p expressers. Secondly, the power for unfavorable prognosis was shown with the combination of low expression of let-7a-2–3p and high expression of miR-188–5p. The patient bearing the combining signatures showed a trend toward shorter OS (*P* = 0.022, Fig. 2C) and EFS (*P* = 0.07, Fig. 2D) than other patients.

**Fig 3:**
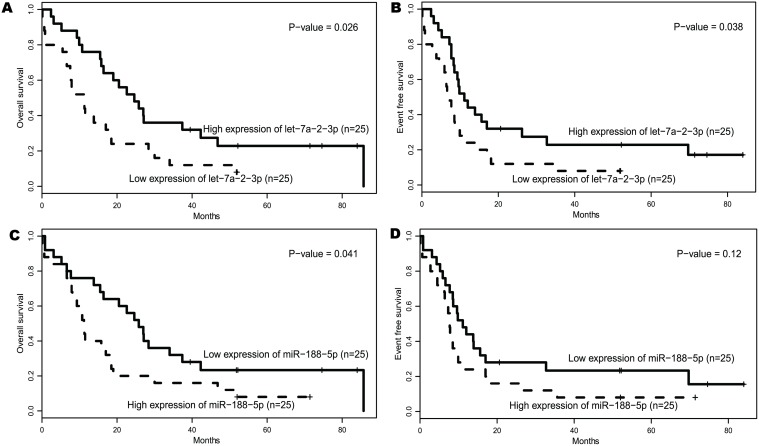
Survival of ELN Intermediate-I CN-AML group according to expression of let-7a-2–3p and miR-188–5p.

According to the mutations of CEBPA, NPM1 and FLT3-ITD, European Leukemia Net (ELN) divided CN-AML patients into ELN Favorable or ELN Intermediate-I genetic categories. 50 of 79 samples used in this paper fell into the ELN Intermediate-I group. Based on these 50 samples, the prognostic impact of these two microRNAs was explored in molecular subsets of CN-AML, which were shown in Fig. 3. Higher let-7a-2–3p expressers had a longer OS (*P* = 0.026, Fig. 3A) and EFS (*P* = 0.038, Fig. 3B) than lower ones, while lower miR-188–5p expressers showed a longer OS (*P* = 0.041, Fig. 3C) and a trend for longer EFS (*P* = 0.12, Fig. 3D) than higher ones. These results further validated the prognostic values of these two microRNAs.

In addition, multivariable analysis was performed to determine the prognostic significance of let-7a-2–3p and miR-188–5p, accompanying other known risk factors. In the multiple model for OS, high let-7a-2–3p expressers had a 54% lower risk of death (*P* = 0.008), and low miR-188–5p expressers showed a 49% lower risk of death (*P* = 0.02). The only other factors associated with longer OS were younger age and FLT3-ITD-negative genotype. In the multiple model of EFS, high let-7a-2–3p expressers had a 52% lower risk of death (*P* = 0.01), and low miR-188–5p expressers showed a 43% lower risk of death (*P* = 0.06). The only other factor associated with longer EFS was younger age and FLT3-ITD negative genotype (See Table 3).

**Table 3:** Multivariable analysis with OS and EFS for the CN-AML patients.

Variable	OS, n = 79, Let-7a-2–3p		EFS, n = 79, Let-7a-2–3p		OS, n = 79, miR-188–5p		EFS, n = 79, miR-188–5p	
	HR(95% CI)	P	HR(95% CI)	P	HR(95% CI)	P	HR(95% CI)	P
microRNA Expression:Let-7a: High *VS* LowmiR-188: Low *VS* High	0.46 (0.27–0.82)	0.008	0.48 (0.27–0.85)	0.01	0.51 (0.29–0.89)	0.02	0.57 (0.33–1.03)	0.06
Age, per 10-y increase	1.42 (1.16–1.76)	0.0007	1.43 (1.15–1.77)	0.001	1.35 (1.11–1.67)	0.003	1.37 (1.11–1.69)	0.004
Sex, male *VS* female	0.69 (0.4–1.2)	0.19	0.68 (0.39–1.18)	0.17	0.68 (0.4–1.16)	0.16	0.67 (0.4–1.16)	0.159
WBC, log10 increase	1.1 (0.69–1.79)	0.68	1.15 (0.71–1.88)	0.56	1.11 (0.69–1.8)	0.66	1.17 (0.72–1.93)	0.52
NPM1, mutated *VS* wild-type	0.66 (0.36–1.19)	0.16	0.61 (0.34–1.12)	0.11	0.62 (0.34–1.12)	0.11	0.63 (0.35–1.14)	0.13
FLT3-ITD, mutated *VS* others	2.2 (1.2–4.1)	0.01	2.5 (1.35–4.61)	0.003	2.18 (1.19–4.0)	0.01	2.37 (1.29–4.35)	0.006
WT1, mutated *VS* wild-type	1.36 (0.53–3.56)	0.52	1.9 (0.72–5.02)	0.19	1.21 (0.47–3.15)	0.69	1.55 (0.6–4.01)	0.37
DNMT3A, mutated *VS* wild-type	1.33(0.77–2.29)	0.3	1.34 (0.75–2.39)	0.32	1.39 (0.81–2.39)	0.23	1.33 (0.76–2.33)	0.32

### Prognostic impact of let-7a-2–3p and miR-188–5p in patients with/without hematopoietic stem cell transplantation

**Fig 4:**
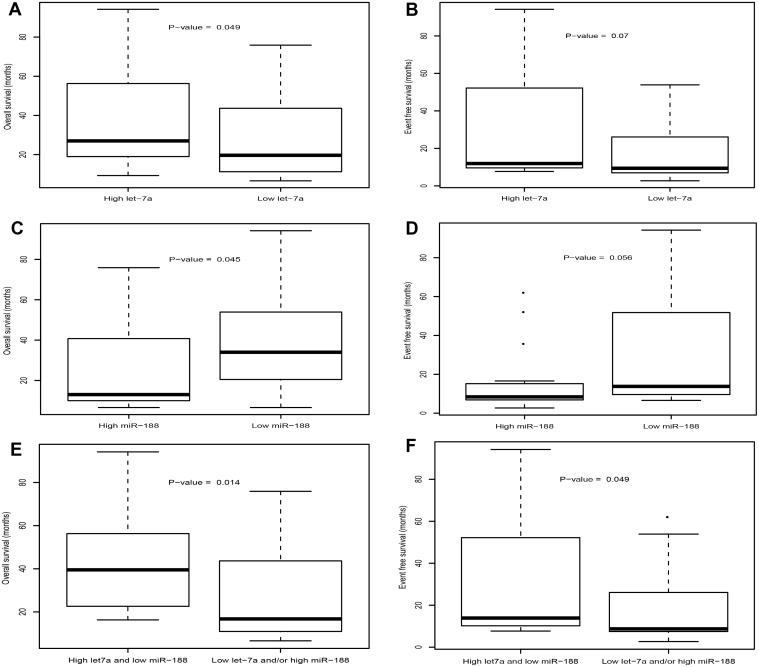
Comparison of OS and EFS according to the expression of let-7a-2–3p and miR-188–5p in patients with HSCT.

Given that the patient cohort included a certain number of patients who had received HSCT, the prognostic impact of these two microRNAs should be further clarified. Patients were divided into two groups, one contained 37 patients with HSCT (6 auto, 31 allo), while the other contained 42 patients without HSCT. Both groups were further partitioned as high/low let-7a-2–3p or miR-188–5p expressers, separately. The distribution of patients with HSCT showed no specificity according to these two microRNAs (See S1 Table). However, OS and EFS showed a significantly difference in all divisions of high/low expression. For patients with HSCT, high let-7a-2–3p expressers showed a longer OS (P = 0.049, Fig. 4A) and a trend for longer EFS (P = 0.07, Fig. 4B), low miR-188–5p expression was associated with a longer OS (P = 0.045, Fig. 4C) and EFS (P = 0.056, Fig. 4D). The combination of high let-7a-2–3p and low miR-188–5p showed a significantly longer OS (P = 0.014, Fig. 4E) and EFS (P = 0.049, Fig. 4F). The prognostic value of these two microRNAs was also further confirmed in patients without HSCT (See S2 Fig., S1 Table). It seemed that high expression of let-7a-2–3p and low expression of miR-188–5p could be used as favorable prognostic biomarkers for CN-AML patients whether they received HSCT or not, independently or in a combined way.

### Gene and microRNA signatures associated with expression levels of let-7a-2–3p

To investigate the underlying biological mechanisms that induce improved outcome for high expression of let-7a-2–3p, we divided the CN-AML patients into different groups according to the median value of let-7a-2–3p expression, and implemented the differential expression analysis for genes and microRNAs on the genome-wide scale.

Comparison between high and low let-7a-2–3p expressers revealed 172 aberrant genes, including 74 up-regulated and 98 down-regulated, as shown in S3 Fig. Higher expression of FOSB and HIST1H4C was accompanied with high let-7a-2–3p expression. The former was previously reported to be sensitive to histone deacetylase inhibitors and associated with favorable outcome in AML [20]. The latter was a known regulator in *NOTCH1*-mediated *NF*-*KB* activity regulation, and closely associated with histone modification [21]. Up-regulation of RASAL2 was also observed, which was recently reported as a tumor and metastasis suppressor [22]. Other up-regulated genes included several members of zinc finger family and small nucleolar family. Furthermore, down-regulated genes associated with high let-7a-2–3p expression included known oncogene JDP2 [23], and several genes in the family of leukocyte immunoglobulin-like receptor (LILRA5/6, LILRB2/3), which might contribute to the adverse prognosis of CN-AML. The full list of differentially expressed genes can be found in supplementary materials (S2 Table).

Here, for the first time, we reported the microRNA expression signatures associated with let-7a-2–3p expression levels in CN-AML. 30 microRNAs were observed to be differentially expressed, including 22 up-regulated and 8 down-regulated signatures, as shown in S4 Fig. Let-7a-2–3p was among the most up-regulated microRNAs in high let-7a-2–3p expressers. miR-100, which induced cell differentiation and better outcome by targeting RBSP3 in AML [24], was also up-regulated. Others included all members of miR-181 family, miR-125a/b, miR-335, which were all previously reported to be associated with higher complete remission (CR) rate and longer OS/EFS in CN-AML[25–27]. One of the 8 down-regulated microRNAs was miR-106a, which was a known oncogenic signature in human T-cell leukemia [28]. The full list of these aberrant microRNAs was shown in supplementary materials (S3 Table).

**Gene and microRNA signatures associated with expression levels of miR-188–5p.** To gain further biological insights associated with favorable prognosis induced by low miR-188–5p expression, we derived the first gene and microRNA expression signatures associated with miR-188–5p expression levels. 34 differentially expressed genes were identified, including 26 up-regulated and 8 down-regulated genes in low miR-188–5p expressers, as shown in S5 Fig. One of the most up-regulated genes was FOSB, which has shown to be associated with favorable outcome in high let-7a-2–3p expressers. Other up-regulated genes included several members of small nucleolar family (SNORD50A, SNORD105, SNORD11B) and zinc finger protein family (ZNF502, ZNF662), which were similar as that in high let-7a-2–3p expressers. The full list of differential genes can be found in supplementary materials (S4 Table).

The first microRNA expression signatures associated with miR-188 expression levels were represented in our study. 77 differentially expressed microRNAs were identified comprising of 18 up-regulated and 59 down-regulated microRNAs in low miR-188 expressers, as shown in S6 Fig. The most up-regulated microRNA was miR-135a, which was recently proved to be a tumor suppressor in epithelial ovarian and regulate HOXA 10 expression [29]. It was also previously report to regulate JAK2 and promote a favorable outcome in classic Hodgkin lymphoma [30]. Other up-regulated microRNAs included miR-335, functioning as a tumor suppressor in pancreatic cancer by targeting OCT4[31]; miR-133a/b, recently reported to inhibit proliferation, migration, invasion and cell cycle by targeting transcription factor SP1 in gastric cancer[32]; all members of miR-181 family, just like in high let-7a-2–3p expressers. Notably, one of the down-regulated microRNAs represented in low miR-188–5p expressers was miR-22, an unfavorable factor targeting TET2 in myelodysplastic syndrome (MDS) and leukemia [33]. The full list of differential microRNAs can be found in supplementary materials (S5 Table).

### Common genes/microRNAs associated with expression of high let-7a-2–3p and low miR-188–5p

Previous results showed that high expression of let-7a-2–3p and low expression of miR188–5p were associated with better prognostic outcome. To further distinctly show their commonly underlying genome-wide expression features, we overlapped the differential genes/microRNAs according to high and low expression of these two microRNAs (See Table 4). Four common genes were found, which were all up-regulated in both high let-7a-2–3p and low miR-188 expressers. 17 shared microRNAs were found, including 10 commonly up-regulated and 7 commonly down-regulated according to high expression of let-7a-2–3p and low expression of miR-188–5p. In these common signatures, several have been proved to be associated with better outcome in AML, such as FOSB, miR-335, miR-181 family members etc. These results further illustrated the possibility of combining let-7a-2–3p and miR-188–5p as prognostic biomarkers for CN-AML patients. Also, other unknown common signatures may aid to reveal the pathogenesis and identify new targets for CN-AML in further studies.

**Table 4:** Common differential genes/microRNAs associated with let-7a-2–3p and miR-188–5p.

Gene Symbol	According to high let-7a-2–3p		According to low miR-188–5p	
	*P*-Value	FC:High/Low	*P*-value	FC:Low/High
SNORD50A	0.00718	1.512576	0.005412	1.524687
FOSB	0.012914	1.699372	0.027542	1.586264
IGJ	0.024882	1.7938	0.046639	1.660184
ZNF502	0.028043	1.628143	0.015428	1.697757
hsa-miR-181d.MIMAT0002821	0.000319056	2.142602773	0.000469785	2.081272036
hsa-miR-181a-2.MIMAT0004558	0.001843197	1.969139207	0.009374555	1.750303828
hsa-miR-181b.MIMAT0000257	0.002259539	1.970094415	0.000770983	2.097643438
hsa-miR-181c.MIMAT0004559	0.002613193	1.92452989	0.00145044	1.987678619
hsa-miR-181a-1.MIMAT0000270	0.036569842	1.726809001	0.004209283	2.113320335
hsa-miR-181a.MIMAT0000256	0.007969811	1.905427863	0.001460825	2.159124058
hsa-miR-335.MIMAT0004703	0.009579031	2.928245868	0.009132969	2.904370331
hsa-miR-335.MIMAT0000765	0.016123333	2.260355584	0.023153169	2.13550789
hsa-miR-125b.MIMAT0000423	0.011682734	2.18596279	0.007880566	2.265970602
hsa-miR-135a.MIMAT0000428	0.041989237	15.10775904	0.036785315	15.51752184
hsa-miR-362.MIMAT0000705	7.79E-05	0.533323567	2.12E-06	0.470050869
hsa-miR-582.MIMAT0003247	0.006783842	0.407669387	0.003404044	0.371317055
hsa-miR-582.MIMAT0004797	0.01043272	0.473921309	0.000976091	0.37183681
hsa-miR-149.MIMAT0000450	0.013208763	0.4614856	0.007721782	0.42985055
hsa-miR-187.MIMAT0000262	0.015363807	0.537003404	0.000527636	0.402208515
hsa-miR-885.MIMAT0004947	0.019571064	0.306167843	0.004242269	0.208654653
hsa-miR-339.MIMAT0000764	0.030419595	0.629686251	0.019395035	0.604786301

## Discussion

Many studies have shown that aberrant expression of microRNA is closely associated with the leukemogenesis, diagnosis and prognosis of AML[34,35]. Several microRNA signatures have been identified to be oncogenetic factors or tumor suppressors, and provide valuable indication for predicting clinical outcome, such as miR-9[36], miR-29b [37]. This paper presents a new endeavor to elucidate the prognostic impact of let-7a-2–3p and miR-188–5p expression in CN-AML.

First, the paper showed higher expression of let-7a-2–3p and lower expression of miR-188–5p were significantly associated with longer OS and EFS in CN-AML patients. Also, their prognostic value was confirmed in ELN genetic categories and validated in multivariable analysis with other known risk factors. Further analysis indicated that these two signatures could be potentially used as favorable prognostic biomarkers for CN-AML patients, whether they received HSCT or not, in an independent or combined way. Thus, these two microRNAs can be used not only as prognostic biomarkers to evaluate the treatment effect, but also potentially as an indicator to direct the choice of therapeutic strategy for transplants in CN-AML.

Based on our searching of existing reports, there were only several papers focusing on the biological and clinical roles of these two microRNAs in various types of tumor research. Only one paper discussed the prognostic impact of let-7a-2, and showed that low expression of let-7a-2 was correlated with poor survivals in lung cancer [38]. On the other hand, there was one paper revealing the potential involvement of miR-188 in human ovarian cell proliferation and apoptosis [39]. To our limited knowledge, this paper was the first one to evaluate the prognostic impact of let-7a-2–3p and miR-188–5p in CN-AML.

**Fig 5:**
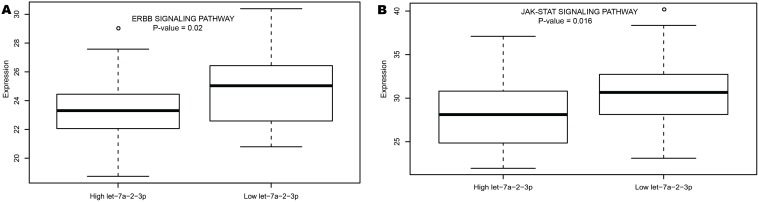
Pathways associated with let-7a-2–3p expression.

Second, based on the genome-wide analysis of gene/microRNA expression profiles, we found many known disease factors were differentially expressed accompanying with the aberrant expression of let-7a-2–3p and miR-188–5p. These genes/microRNAs involved in many important genetic and epigenetic processes, such as cell proliferation, differentiation, immune response, methylation and acetylation, which can potentially reveal the underlying mechanisms why let-7a-2–3p and miR-188 functioned. In addition, pathway data from MSigDB [40] was used to show the expression alternation associated with microRNA expression levels. In high let-7a-2–3p expressers, several known pathways that indicate unfavorable outcome were significantly down-regulated (S6 Table), including ERBB Signaling pathway [41] (*P* = 0.02, Fig. 5A), JAK-STAT Signaling pathway [42] (P = 0.016, Fig. 5B).

Interestingly, some common microRNAs previously verified to be associated with better outcome were up-regulated in both high let-7a-2–3p expressers and low miR-188–5p expressers, including miR-335, miR-125b, miR-135a and all members of miR-181 family. Common up-regulated gene signatures included FOSB, members of zinc finger family and small nucleolar family in both high let-7a-2–3p expressers and low miR-188–5p expressers. FOSB has been verified to be a cofactor for JUN family to regulate proliferation and differentiation. The zinc finger family and small nucleolar family may be new disease factors for CN-AML. These findings indicated the possibly common function mechanisms for let-7a-2–3p and miR-188, and provided more support for the combined use for these two signatures in prognostic evaluation for CN-AML patients.

In conclusion, the paper identified high expression of let-7a-2–3p and low expression of miR-188–5p as two potential prognostic biomarkers for improved outcome in CN-AML patients whether they received HSCT or not. The relatively high accuracy of high-throughput sequencing data provided strong support for these discoveries. Limited by the relatively small sample number, more rigorous checkout should be carried out before their application in risk-stratification of CN-AML patients. These results presented valuable exploration about the function and clinical aid of let-7a-2–3p and miR-188–5p. Their prognostic value and associated aberrant expressed genes/microRNAs can improve the understanding of the internal mechanisms of leukemogesis and prompt the designing of new therapy strategies for CN-AML patients.

## Supporting Information

S1 Fig Flowchart for the selection of let-7a-2–3p and miR-188–5p. (EPS)

S2 Fig Comparison of OS and EFS according to the expression of let-7a-2–3p and miR-188–5p in patients without HSCT. (EPS)

S3 Fig Heatmap of differential genes associated with the expression of let-7a-2–3p. (EPS)

S4 Fig Heatmap of differential microRNAs associated with the expression of let-7a-2–3p. (EPS)

S5 Fig Heatmap of differential genes associated with the expression of mir-188–5p. (EPS)

S6 Fig Heatmap of differential microRNAs associated with the expression of mir-188–5p. (EPS)

S7 Fig Barplot of miR-135a expression according to miR-188. (EPS)

S8 Fig Boxplot of let-7a and miR-188 expression in different cytogenetic groups. (EPS)

S9 Fig Comparison of OS and EFS according to expression of let-7a-2–3p and miR-188–5p in other cytogenetic groups. (EPS)

S1 Table Treatment response according to let-7a-2–3p and miR-188–5p expression for patients with/without HSCT. (DOC)

S2 Table Differentially expressed genes according to high let-7a-2–3p expression. (DOC)

S3 Table Differentially expressed microRNAs according to high let-7a-2–3p expression. (DOC)

S4 Table Differentially expressed genes according to low mir-188–5p expression. (DOC)

S5 Table Differentially expressed microRNAs according to low mir-188–5p expression. (DOC)

S6 Table Pathways associated with let-7a-2–3p expression levels. (DOC)
