# Supplementary material for: Identification of let-7a-2-3p or/and miR-188-5p as Prognostic Biomarkers in Cytogenetically Normal Acute Myeloid Leukemia
Source: PLoS One. 2015 Feb 3;10(2):e0118099. doi: 10.1371/journal.pone.0118099 (PMC4315415; doi:10.1371/journal.pone.0118099)
Supplement: S1 Table — (DOC) [file pone.0118099.s010.doc]

**Table S1. Treatment response according to let-7a-2-3p and miR-188-5p expression for patients with/without HSCT**

|  | **High let-7a** | **Low let-7a** | **p** | **High miR-188** | **Low miR-188,** | **p** | **High let-7a and low miR-188** | **High let-7a and/or low miR-188** | **p** |
| --- | --- | --- | --- | --- | --- | --- | --- | --- | --- |
| **With transplant, no** | 17 | 20 | 0.65 | 16 | 21 |  | 13 | 24 | 1 |
| **OS, months** |  |  | 0.049 |  |  | 0.045 |  |  | 0.013 |
| Median | 27 | 19.65 |  | 13 | 34 |  | 39.5 | 16.75 |  |
| Range | 9.3-94.2 | 6.6-75.9 |  | 6.6-75.9 | 6.6-94.2 |  | 16.3-94.2 | 6.6-75.9 |  |
| **EFS, months** |  |  | 0.07 |  |  | 0.056 |  |  | 0.049 |
| Median | 11.9 | 9.4 |  | 8.4 | 13.8 |  | 13.9 | 8.75 |  |
| Range | 7.7-94.2 | 2.7-53.9 |  | 2.7-62 | 6.6-94.2 |  | 7.7-94.2 | 2.7-62 |  |
| Without transplants, no | 22 | 20 | 0.6 | 23 | 19 |  | 14 | 28 | 1 |
| **OS, months** | 22 | 20 | 0.002 |  |  | 0.015 |  |  | <0.001 |
| Median | 22.3 | 6.9 |  | 9.9 | 15.5 |  | 27.1 | 7.9 |  |
| Range | 0.4-84.5 | 0.1-42.1 |  | 0.1-46.8 | 0.4-84.5 |  | 0.4-84.5 | 0.1-46.8 |  |
| **EFS, months** |  |  | 0.01 |  |  | 0.021 |  |  | 0.002 |
| Median | 12 | 5.2 |  | 7.8 | 8.0 |  | 15.6 | 6.9 |  |
| Range | 0.4-84.5 | 0.1-42.1 |  | 0.1-42.1 | 0.4-84.5 |  | 0.4-84.5 | 0.1-42.1 |  |
