# Supplementary material for: Identification of let-7a-2-3p or/and miR-188-5p as Prognostic Biomarkers in Cytogenetically Normal Acute Myeloid Leukemia
Source: PLoS One. 2015 Feb 3;10(2):e0118099. doi: 10.1371/journal.pone.0118099 (PMC4315415; doi:10.1371/journal.pone.0118099)
Supplement: S2 Table — (DOC) [file pone.0118099.s011.doc]

**Table S2. Differentially** expressed genes according to high let-7a-2-3p expression

| **Gene symbol** | **P-value** | **Fold change: High/Low** |
| --- | --- | --- |
| DENND5B | 6.18E-05 | 1.798734 |
| KDELR3 | 0.000321 | 1.561834 |
| SNORD81 | 0.000841 | 1.568867 |
| SNORD78 | 0.000918 | 1.610048 |
| SNORD77 | 0.00101 | 1.585713 |
| CCDC65 | 0.001211 | 1.512082 |
| SNORD85 | 0.001682 | 1.876873 |
| SLC35F2 | 0.001698 | 1.576414 |
| ZNF714 | 0.001942 | 1.532036 |
| LEPREL4 | 0.002369 | 1.665571 |
| SNORD75 | 0.003272 | 1.568274 |
| GLCCI1 | 0.003395 | 1.58721 |
| SNORD50B | 0.00368 | 1.537178 |
| NEDD4 | 0.004105 | 1.518438 |
| HDGFRP3 | 0.004376 | 2.97701 |
| TXNRD3IT1 | 0.005112 | 1.536738 |
| PBK | 0.005504 | 1.939164 |
| RASAL2 | 0.005545 | 1.766416 |
| LTBP1 | 0.005862 | 3.628377 |
| ZNF404 | 0.006838 | 1.597784 |
| SNORD50A | 0.00718 | 1.512576 |
| LRP12 | 0.007642 | 1.797863 |
| CHPF | 0.007659 | 1.701712 |
| SNORD121A | 0.007876 | 1.550112 |
| LHFP | 0.00919 | 2.060053 |
| CENPV | 0.009908 | 1.593 |
| ASPM | 0.010275 | 1.572565 |
| SRSF12 | 0.010775 | 1.52655 |
| ADAM6 | 0.011181 | 2.299929 |
| ZNF627 | 0.01141 | 1.537329 |
| ZNF681 | 0.012448 | 1.533145 |
| HYMAI | 0.012808 | 1.561692 |
| FOSB | 0.012914 | 1.699372 |
| SNORA23 | 0.013926 | 1.650311 |
| SNORA14B | 0.014333 | 1.507417 |
| PDZD8 | 0.014361 | 1.68709 |
| PIF1 | 0.01515 | 1.805689 |
| KIT | 0.015169 | 1.730503 |
| TNFRSF17 | 0.015293 | 1.606696 |
| ZNF486 | 0.015914 | 1.633113 |
| CDC20 | 0.016677 | 1.541405 |
| TMEM22 | 0.017082 | 1.721571 |
| GPR125 | 0.018559 | 1.531319 |
| GPHN | 0.01878 | 1.537223 |
| VANGL1 | 0.020153 | 1.632738 |
| TAS2R4 | 0.020228 | 1.572864 |
| ARHGAP32 | 0.020857 | 1.948001 |
| C1R | 0.021733 | 2.025886 |
| RHOBTB3 | 0.02278 | 1.619197 |
| DEPDC1 | 0.022792 | 1.560896 |
| DCBLD2 | 0.023412 | 1.617145 |
| IGJ | 0.024882 | 1.7938 |
| B3GALNT1 | 0.026587 | 1.611892 |
| DEGS2 | 0.026924 | 1.619278 |
| LEPR | 0.02731 | 1.83179 |
| PDZD2 | 0.027747 | 1.655771 |
| ZNF502 | 0.028043 | 1.628143 |
| PRICKLE1 | 0.03265 | 1.601382 |
| CYP7B1 | 0.032709 | 1.987549 |
| RAB23 | 0.033363 | 1.508613 |
| SPINK4 | 0.033467 | 1.527533 |
| SDPR | 0.033508 | 2.40587 |
| LPAR4 | 0.033907 | 1.676806 |
| FKBP14 | 0.03409 | 1.51985 |
| HIST1H4C | 0.035097 | 1.625377 |
| SH3D19 | 0.035177 | 1.506191 |
| TAS2R31 | 0.03702 | 1.510814 |
| TNIK | 0.037084 | 1.889452 |
| MEX3B | 0.038656 | 1.926702 |
| TRIM6 | 0.039379 | 1.53709 |
| CRISPLD1 | 0.043705 | 1.658539 |
| SNORD26 | 0.047671 | 1.501509 |
| C1S | 0.049302 | 1.84661 |
| CTDSPL | 0.04959 | 1.745215 |
| DNASE2 | 0.00117 | 0.640426 |
| RPP25 | 0.001267 | 0.653401 |
| NAAA | 0.002211 | 0.632622 |
| SLC2A6 | 0.005614 | 0.538545 |
| SLC22A18 | 0.006769 | 0.659765 |
| BAIAP3 | 0.007517 | 0.625871 |
| CD14 | 0.008101 | 0.352136 |
| ANXA2 | 0.008622 | 0.579979 |
| PLA2G16 | 0.008781 | 0.363826 |
| TYROBP | 0.009756 | 0.595497 |
| SLC46A2 | 0.009827 | 0.535064 |
| CLEC7A | 0.010618 | 0.458287 |
| SAT1 | 0.010645 | 0.552611 |
| CFP | 0.010824 | 0.56815 |
| SERPINB9 | 0.011158 | 0.565883 |
| SLC15A3 | 0.012668 | 0.500058 |
| ALOX5 | 0.012901 | 0.504767 |
| TNFSF12 | 0.012994 | 0.531947 |
| BRI3 | 0.013552 | 0.664199 |
| SECTM1 | 0.013836 | 0.407799 |
| DOK2 | 0.014957 | 0.594067 |
| COTL1 | 0.014961 | 0.620636 |
| PIK3R2 | 0.015096 | 0.577781 |
| CTSB | 0.015543 | 0.615571 |
| NACC2 | 0.016076 | 0.616448 |
| IFI30 | 0.016113 | 0.489692 |
| TMEM150B | 0.016458 | 0.513163 |
| TYMP | 0.017319 | 0.44923 |
| FCN1 | 0.017673 | 0.454007 |
| C17orf91 | 0.017801 | 0.593108 |
| TBC1D9 | 0.019208 | 0.529542 |
| S100A11 | 0.019285 | 0.588299 |
| SGSH | 0.019733 | 0.565206 |
| ASGR2 | 0.020282 | 0.478401 |
| LDLR | 0.02057 | 0.587623 |
| GLIPR2 | 0.020617 | 0.668738 |
| KLF11 | 0.020784 | 0.638469 |
| GPA33 | 0.020883 | 0.483346 |
| SLC26A11 | 0.021183 | 0.621306 |
| FTL | 0.021372 | 0.660106 |
| SLC7A7 | 0.021974 | 0.456041 |
| CARD16 | 0.022069 | 0.652784 |
| UPP1 | 0.022747 | 0.546562 |
| MT2A | 0.023048 | 0.500075 |
| PLD2 | 0.023213 | 0.597824 |
| SIGLEC7 | 0.023526 | 0.534085 |
| GIMAP4 | 0.024777 | 0.425889 |
| NRBP2 | 0.025127 | 0.634057 |
| SYTL3 | 0.025609 | 0.616153 |
| STX11 | 0.025663 | 0.612385 |
| RASSF4 | 0.027669 | 0.611574 |
| TPPP3 | 0.028182 | 0.393384 |
| CLEC4A | 0.028456 | 0.585093 |
| ODF3B | 0.028885 | 0.468466 |
| GAA | 0.029257 | 0.657941 |
| ANXA5 | 0.029987 | 0.571185 |
| FGR | 0.030097 | 0.603964 |
| SLC31A2 | 0.030315 | 0.643911 |
| CES4A | 0.030332 | 0.589339 |
| NOD2 | 0.031049 | 0.601159 |
| KCTD12 | 0.031355 | 0.558512 |
| LY96 | 0.031524 | 0.540914 |
| SERPINA1 | 0.032193 | 0.501114 |
| PLBD1 | 0.032358 | 0.331546 |
| APOL3 | 0.034175 | 0.66669 |
| C1orf162 | 0.034236 | 0.632441 |
| AMICA1 | 0.034516 | 0.600502 |
| MS4A6A | 0.034622 | 0.599709 |
| DPYSL2 | 0.034927 | 0.600767 |
| MT1A | 0.036059 | 0.530473 |
| JDP2 | 0.03623 | 0.660456 |
| GBP2 | 0.036528 | 0.635822 |
| LILRB3 | 0.03786 | 0.571406 |
| FCGRT | 0.038977 | 0.665193 |
| FTH1 | 0.039554 | 0.669564 |
| FCGR2A | 0.039594 | 0.547826 |
| S100A10 | 0.039925 | 0.579777 |
| LILRB2 | 0.039969 | 0.519124 |
| LGALS3 | 0.040195 | 0.566177 |
| SIGLEC9 | 0.040764 | 0.567151 |
| TREM1 | 0.041224 | 0.587855 |
| CPVL | 0.041445 | 0.610219 |
| TLR5 | 0.042155 | 0.576892 |
| CTSH | 0.042623 | 0.613905 |
| CD48 | 0.043168 | 0.58129 |
| LRRK2 | 0.04374 | 0.525098 |
| FLVCR2 | 0.04385 | 0.57296 |
| CTSS | 0.044087 | 0.589313 |
| NPL | 0.045633 | 0.503396 |
| LILRA5 | 0.045801 | 0.471152 |
| LILRA6 | 0.045861 | 0.556489 |
| IGSF6 | 0.045863 | 0.621803 |
| HLA-DQA2 | 0.046761 | 0.432497 |
| C4orf48 | 0.046863 | 0.628824 |
| TCN2 | 0.047115 | 0.445835 |
| SPINT1 | 0.048434 | 0.654929 |
| RBM47 | 0.049121 | 0.609959 |
| GPBAR1 | 0.049652 | 0.480847 |
