# Supplementary material for: Identification of let-7a-2-3p or/and miR-188-5p as Prognostic Biomarkers in Cytogenetically Normal Acute Myeloid Leukemia
Source: PLoS One. 2015 Feb 3;10(2):e0118099. doi: 10.1371/journal.pone.0118099 (PMC4315415; doi:10.1371/journal.pone.0118099)
Supplement: S3 Table — (DOC) [file pone.0118099.s012.doc]

**Table S3. Differentially** expressed microRNAs according to high let-7a-2-3p expression

| **microRNA symbol** | **P-value** | **Fold change: High/Low** |
| --- | --- | --- |
| hsa-mir-181d.MIMAT0002821 | 0.000319056 | 2.142602773 |
| hsa-let-7a-2.MIMAT0010195 | 0.000353176 | 29.4615683 |
| hsa-let-7e.MIMAT0000066 | 0.001502812 | 1.503740788 |
| hsa-mir-181a-2.MIMAT0004558 | 0.001843197 | 1.969139207 |
| hsa-mir-181b.MIMAT0000257 | 0.002259539 | 1.970094415 |
| hsa-mir-181c.MIMAT0004559 | 0.002613193 | 1.92452989 |
| hsa-mir-219-1.MIMAT0004567 | 0.003144807 | 1.659808318 |
| hsa-mir-100.MIMAT0000098 | 0.007153053 | 13.25255861 |
| hsa-mir-181a.MIMAT0000256 | 0.007969811 | 1.905427863 |
| hsa-mir-335.MIMAT0004703 | 0.009579031 | 2.928245868 |
| hsa-mir-125b.MIMAT0000423 | 0.011682734 | 2.18596279 |
| hsa-mir-194-2.MIMAT0004671 | 0.013849518 | 1.619668365 |
| hsa-let-7e.MIMAT0004485 | 0.015418946 | 2.058561787 |
| hsa-mir-335.MIMAT0000765 | 0.016123333 | 2.260355584 |
| hsa-mir-99b.MIMAT0004678 | 0.020356855 | 1.993207105 |
| hsa-mir-125a.MIMAT0004602 | 0.022723711 | 2.056853899 |
| hsa-mir-139.MIMAT0000250 | 0.029412499 | 2.352590803 |
| hsa-mir-181a-1.MIMAT0000270 | 0.036569842 | 1.726809001 |
| hsa-mir-99b.MIMAT0000689 | 0.03725519 | 1.893810835 |
| hsa-mir-135a.MIMAT0000428 | 0.041989237 | 15.10775904 |
| hsa-mir-25.MIMAT0004498 | 0.046905176 | 1.615320425 |
| hsa-mir-125a.MIMAT0000443 | 0.047868423 | 1.888452282 |
| hsa-mir-362.MIMAT0000705 | 7.79E-05 | 0.533323567 |
| hsa-mir-582.MIMAT0003247 | 0.006783842 | 0.407669387 |
| hsa-mir-582.MIMAT0004797 | 0.01043272 | 0.473921309 |
| hsa-mir-149.MIMAT0000450 | 0.013208763 | 0.4614856 |
| hsa-mir-187.MIMAT0000262 | 0.015363807 | 0.537003404 |
| hsa-mir-106a.MIMAT0004517 | 0.018299467 | 0.342436159 |
| hsa-mir-885.MIMAT0004947 | 0.019571064 | 0.306167843 |
| hsa-mir-339.MIMAT0000764 | 0.030419595 | 0.629686251 |
