# Supplementary material for: Identification of let-7a-2-3p or/and miR-188-5p as Prognostic Biomarkers in Cytogenetically Normal Acute Myeloid Leukemia
Source: PLoS One. 2015 Feb 3;10(2):e0118099. doi: 10.1371/journal.pone.0118099 (PMC4315415; doi:10.1371/journal.pone.0118099)
Supplement: S4 Table — (DOC) [file pone.0118099.s013.doc]

**Table S4. Differentially** expressed genes according to low mir-188-5p expression

| Gene symbol | P-value | Fold change: Low/High |
| --- | --- | --- |
| SNORD50A | 0.005412 | 1.524687 |
| PYGM | 0.008033 | 1.650911 |
| CA2 | 0.009995 | 1.831582 |
| C16orf74 | 0.010415 | 1.552759 |
| SLAIN1 | 0.010756 | 1.869713 |
| AMMECR1 | 0.013089 | 1.661231 |
| ZNF502 | 0.015428 | 1.697757 |
| GPR141 | 0.019289 | 1.603505 |
| PON2 | 0.019482 | 1.514528 |
| SNORD105 | 0.020841 | 1.578168 |
| EPHB4 | 0.026518 | 1.669729 |
| F8A1 | 0.027311 | 1.664957 |
| FOSB | 0.027542 | 1.586264 |
| TAS2R41 | 0.027866 | 1.641755 |
| PIP5K1B | 0.028142 | 1.681383 |
| TPBG | 0.031026 | 1.925911 |
| C6orf192 | 0.032167 | 1.557199 |
| ZNF662 | 0.035139 | 1.690256 |
| RHD | 0.03545 | 1.684725 |
| QPCT | 0.03817 | 2.798953 |
| MGST1 | 0.039173 | 1.505206 |
| HMGN5 | 0.043375 | 1.62298 |
| SNORD11B | 0.044568 | 1.619247 |
| IGJ | 0.046639 | 1.660184 |
| LOC220594 | 0.046649 | 1.732303 |
| FAM71F2 | 0.049661 | 1.527925 |
| TMEM51 | 0.002846 | 0.506539 |
| BAIAP2 | 0.003621 | 0.636513 |
| GIMAP6 | 0.003732 | 0.612342 |
| C9orf9 | 0.020875 | 0.658006 |
| EDA2R | 0.027812 | 0.577726 |
| MGC12982 | 0.038235 | 0.649437 |
| LGALS3BP | 0.042658 | 0.628722 |
| NRN1L | 0.044623 | 0.63715 |
