# Supplementary material for: Identification of let-7a-2-3p or/and miR-188-5p as Prognostic Biomarkers in Cytogenetically Normal Acute Myeloid Leukemia
Source: PLoS One. 2015 Feb 3;10(2):e0118099. doi: 10.1371/journal.pone.0118099 (PMC4315415; doi:10.1371/journal.pone.0118099)
Supplement: S5 Table — (DOC) [file pone.0118099.s014.doc]

**Table S5. Differentially** expressed microRNAs according to low mir-188-5p expression

| **microRNA symbol** | **P-value** | **Fold change: Low/High** |
| --- | --- | --- |
| hsa-mir-181d.MIMAT0002821 | 0.000469785 | 2.081272036 |
| hsa-mir-181b.MIMAT0000257 | 0.000770983 | 2.097643438 |
| hsa-mir-181c.MIMAT0004559 | 0.00145044 | 1.987678619 |
| hsa-mir-181a.MIMAT0000256 | 0.001460825 | 2.159124058 |
| hsa-mir-181c.MIMAT0000258 | 0.002508822 | 1.981197521 |
| hsa-mir-181a-1.MIMAT0000270 | 0.004209283 | 2.113320335 |
| hsa-mir-125b.MIMAT0000423 | 0.007880566 | 2.265970602 |
| hsa-mir-1468.MIMAT0006789 | 0.009018266 | 1.757166298 |
| hsa-mir-196b.MIMAT0001080 | 0.009025377 | 1.548380466 |
| hsa-mir-335.MIMAT0004703 | 0.009132969 | 2.904370331 |
| hsa-mir-181a-2.MIMAT0004558 | 0.009374555 | 1.750303828 |
| hsa-mir-766.MIMAT0003888 | 0.012849033 | 1.571740922 |
| hsa-mir-1275.MIMAT0005929 | 0.015699365 | 2.098539089 |
| hsa-mir-335.MIMAT0000765 | 0.023153169 | 2.13550789 |
| hsa-mir-133b.MIMAT0000770 | 0.024928742 | 2.113577846 |
| hsa-mir-1.MIMAT0000416 | 0.028266594 | 1.932004052 |
| hsa-mir-133a.MIMAT0000427 | 0.034694445 | 1.875944457 |
| hsa-mir-135a.MIMAT0000428 | 0.036785315 | 15.51752184 |
| hsa-mir-188.MIMAT0000457 | 4.67E-16 | 0.209732869 |
| hsa-mir-660.MIMAT0003338 | 4.30E-07 | 0.417266503 |
| hsa-mir-362.MIMAT0000705 | 2.12E-06 | 0.470050869 |
| hsa-mir-500.MIMAT0002871 | 2.73E-06 | 0.482024398 |
| hsa-mir-22.MIMAT0004495 | 3.00E-06 | 0.510024776 |
| hsa-mir-362.MIMAT0004683 | 3.22E-06 | 0.375503884 |
| hsa-mir-532.MIMAT0002888 | 6.55E-06 | 0.529446416 |
| hsa-mir-500.MIMAT0004773 | 8.93E-06 | 0.480862366 |
| hsa-mir-532.MIMAT0004780 | 1.10E-05 | 0.443266011 |
| hsa-mir-501.MIMAT0004774 | 2.19E-05 | 0.471520358 |
| hsa-mir-501.MIMAT0002872 | 4.97E-05 | 0.495689375 |
| hsa-mir-22.MIMAT0000077 | 9.89E-05 | 0.564432867 |
| hsa-mir-502.MIMAT0004775 | 0.000145263 | 0.597685258 |
| hsa-mir-1249.MIMAT0005901 | 0.000165409 | 0.546658941 |
| hsa-mir-21.MIMAT0004494 | 0.000407612 | 0.468926793 |
| hsa-mir-193a.MIMAT0000459 | 0.000409864 | 0.367124878 |
| hsa-mir-187.MIMAT0000262 | 0.000527636 | 0.402208515 |
| hsa-mir-29b.MIMAT0000100 | 0.00054772 | 0.621541556 |
| hsa-mir-877.MIMAT0004949 | 0.00063757 | 0.555105798 |
| hsa-mir-33a.MIMAT0000091 | 0.000638299 | 0.41641716 |
| hsa-mir-33a.MIMAT0004506 | 0.000700598 | 0.596619536 |
| hsa-mir-185.MIMAT0004611 | 0.000956159 | 0.643880707 |
| hsa-mir-582.MIMAT0004797 | 0.000976091 | 0.37183681 |
| hsa-mir-365.MIMAT0000710 | 0.001245136 | 0.491731105 |
| hsa-mir-193a.MIMAT0004614 | 0.001282717 | 0.562605578 |
| hsa-mir-24-2.MIMAT0004497 | 0.001810493 | 0.65457909 |
| hsa-mir-708.MIMAT0004927 | 0.003032071 | 0.193965098 |
| hsa-mir-324.MIMAT0000761 | 0.003136107 | 0.660449842 |
| hsa-mir-582.MIMAT0003247 | 0.003404044 | 0.371317055 |
| hsa-mir-708.MIMAT0004926 | 0.003671302 | 0.302319426 |
| hsa-mir-885.MIMAT0004947 | 0.004242269 | 0.208654653 |
| hsa-mir-574.MIMAT0003239 | 0.004312205 | 0.578726286 |
| hsa-mir-145.MIMAT0004601 | 0.004527763 | 0.469930375 |
| hsa-mir-590.MIMAT0003258 | 0.005013117 | 0.649252637 |
| hsa-mir-29c.MIMAT0000681 | 0.006292097 | 0.615084169 |
| hsa-mir-33b.MIMAT0003301 | 0.00637642 | 0.457557465 |
| hsa-mir-511.MIMAT0002808 | 0.006919834 | 0.153627415 |
| hsa-mir-7.MIMAT0000252 | 0.007434974 | 0.521794763 |
| hsa-mir-149.MIMAT0000450 | 0.007721782 | 0.42985055 |
| hsa-mir-34a.MIMAT0000255 | 0.009148147 | 0.591417317 |
| hsa-mir-193b.MIMAT0004767 | 0.009573821 | 0.331476185 |
| hsa-mir-21.MIMAT0000076 | 0.011859273 | 0.649463717 |
| hsa-mir-151.MIMAT0000757 | 0.011995321 | 0.38969252 |
| hsa-mir-153.MIMAT0000439 | 0.012041751 | 0.215202762 |
| hsa-mir-143.MIMAT0004599 | 0.012693902 | 0.388143637 |
| hsa-mir-145.MIMAT0000437 | 0.015294758 | 0.558184651 |
| hsa-mir-548o.MIMAT0005919 | 0.018423994 | 0.634445635 |
| hsa-mir-339.MIMAT0000764 | 0.019395035 | 0.604786301 |
| hsa-mir-301b.MIMAT0004958 | 0.019572278 | 0.503344328 |
| hsa-mir-152.MIMAT0000438 | 0.020414068 | 0.59362969 |
| hsa-mir-143.MIMAT0000435 | 0.020806124 | 0.413633893 |
| hsa-mir-497.MIMAT0002820 | 0.021426697 | 0.439640457 |
| hsa-mir-212.MIMAT0000269 | 0.024314665 | 0.61658253 |
| hsa-mir-1226.MIMAT0005577 | 0.024856918 | 0.641179652 |
| hsa-mir-301a.MIMAT0000688 | 0.028057639 | 0.608641031 |
| hsa-mir-195.MIMAT0000461 | 0.031825752 | 0.430045202 |
| hsa-mir-1237.MIMAT0005592 | 0.04047027 | 0.647104158 |
| hsa-mir-148a.MIMAT0004549 | 0.043126172 | 0.591500584 |
| hsa-mir-151.MIMAT0004697 | 0.043947535 | 0.403764625 |
