# Supplementary material for: Identification of let-7a-2-3p or/and miR-188-5p as Prognostic Biomarkers in Cytogenetically Normal Acute Myeloid Leukemia
Source: PLoS One. 2015 Feb 3;10(2):e0118099. doi: 10.1371/journal.pone.0118099 (PMC4315415; doi:10.1371/journal.pone.0118099)
Supplement: S6 Table — (DOC) [file pone.0118099.s015.doc]

**Table S6.** Pathways associated with let-7a-2-3p expression levels

| **Pathway name** | **According to high let-7a-2-3p** | |
| --- | --- | --- |
|  | **Regulation** | ***P*-Value** |
| KEGG_PENTOSE_PHOSPHATE_PATHWAY | Down | 0.028 |
| KEGG_FRUCTOSE_AND_MANNOSE_METABOLISM | Down | 0.025 |
| KEGG_GALACTOSE_METABOLISM | Down | 0.03 |
| KEGG_O_GLYCAN_BIOSYNTHESIS | Up | 0.008 |
| KEGG_ETHER_LIPID_METABOLISM | Up | 0.029 |
| KEGG_ARACHIDONIC_ACID_METABOLISM | Down | 0.018 |
| KEGG_DRUG_METABOLISM_OTHER_ENZYMES | Down | 0.026 |
| KEGG_ERBB_SIGNALING_PATHWAY | Down | 0.02 |
| KEGG_CALCIUM_SIGNALING_PATHWAY | Down | 0.027 |
| KEGG_LYSOSOME | Down | 0.04 |
| KEGG_ENDOCYTOSIS | Down | 0.041 |
| KEGG_TGF_BETA_SIGNALING_PATHWAY | Down | 0.065 |
| KEGG_VEGF_SIGNALING_PATHWAY | Down | 0.061 |
| KEGG_FOCAL_ADHESION | Down | 0.063 |
| KEGG_ADHERENS_JUNCTION | Down | 0.058 |
| KEGG_TIGHT_JUNCTION | Down | 0.057 |
| KEGG_JAK_STAT_SIGNALING_PATHWAY | Down | 0.016 |
| KEGG_FC_EPSILON_RI_SIGNALING_PATHWAY | Down | 0.061 |
| KEGG_FC_GAMMA_R_MEDIATED_PHAGOCYTOSIS | Down | 0.033 |
| KEGG_NEUROTROPHIN_SIGNALING_PATHWAY | Down | 0.045 |
| KEGG_LONG_TERM_DEPRESSION | Down | 0.023 |
| KEGG_REGULATION_OF_ACTIN_CYTOSKELETON | Down | 0.056 |
| KEGG_TYPE_II_DIABETES_MELLITUS | Down | 0.038 |
| KEGG_RENAL_CELL_CARCINOMA | Down | 0.028 |
| KEGG_PANCREATIC_CANCER | Down | 0.052 |
| KEGG_ENDOMETRIAL_CANCER | Down | 0.036 |
| KEGG_CHRONIC_MYELOID_LEUKEMIA | Down | 0.048 |
